# Meditation-based clinical study to determine the correlation of quantitative electroencephalogram (qEEG) and 24-hour EEG activity

**DOI:** 10.1097/MD.0000000000012557

**Published:** 2018-10-26

**Authors:** Moon Joo Cheong, Sung-Eun Son, Hyung Won Kang, Youngseop Lee, Kwang-Ho Bae, Yeonseok Kang, Eun Mi Lee, Go-Eun Lee, Joo-Hee Seo, Hee-Wook Weon, Ji-Yoon Lee, Yeoung-Su Lyu

**Affiliations:** a Education Graduate of Hanyang University, Seoul; b Department of Korean Neuropsychiatry Medicine, College of Korean Medicine, Wonkwang University, 460 Iksan-daero, Iksan-si, Jeollabuk-do; c Department of Korean Neuropsychiatry Medicine & Inam Neuroscience Research Center, Wonkwang University Sanbon Hospital, Gunpo; d Future Medicine Division, Korea Institute of Oriental Medicine, 1672 Yuseong-daero, Yuseong-gu, Daejeon; e Department of Medical History, College of Korean Medicine, Wonkwang University; f Division of Cardiology, Department of Internal Medicine, Wonkwang University Sanbon, Hospital, Gyeonggi-do; g Department of Oriental Rehabilitation Medicine, Korean National Rehabilitation; h National Medical Center, 245 Uljiro, Jung-gu; i Seoul University of Buddhism; j Department of Psychology, Graduate school of Ewha Woman's University, Seoul, Republic of Korea.

**Keywords:** 24-hour Holter monitor, activity ECG, meditation and mindfulness, qEEG, randomized controlled trial, stress and resource, study protocol

## Abstract

**Background:**

The heart continuously transmits information to the cerebrum during each pulse, and influences information processing such as perception, cognition, and emotion, which are processed in the cerebrum. This is the basis for the theory of oriental medicine widely used in psychiatric medicine and clinical practice, so-called Simjushinji (heart and brain) theory, that the heart controls the mind. The present study aims to analyze the correlation between heart and brain function by 24-hour active electrocardiogram and quantitative electroencephalogram (EEG) measurement under meditation.

**Methods:**

This randomized, controlled, assessor-blinded, 2-armed, parallel, multicenter clinical trial will analyze a total of 50 subjects, including 25 each for the test group and the active control group. Subjects will be randomly allocated to the test group (performing resource mindfulness) and the control group (performing stress mindfulness) in a 1:1 ratio. The clinical trial consists of 3 stages. The first and third stages are stable states. The second stage is divided into the test and active comparator groups. Quantitative EEG (qEEG) measurements at stages 1 and 3 will be recorded for 10 minutes; measurements at stage 2 will be recorded for 20 minutes with the eyes closed. The 24-hour Holter Monitoring and heart rate variability will be evaluated at each stage. Before the beginning of stage 3, subjects will complete the questionnaires. The primary outcome will be analyzed by independent *t* tests of both groups.

**Discussion:**

Scientific studies based on clinical epistemology are expected to serve as a basis for sustainable medical services in the field of psychiatric medicine in Korea. HRV, blood pressure index, and biometric index in qEEG, as determined by 24-hour Holter monitoring, will complement quantitative biomarkers and be useful in various fields.

## 1 Background

Brain and cardiovascular diseases contribute to the highest percentage of single-cause deaths (12.8%) worldwide. According to a 2017 report by the World Health Organization, 23.5 million people worldwide die of brain and cardiovascular diseases each year. In the West and in the Orient, a primary cause of these deaths was reported as mental stress.^[1,2]^ From the perspective of oriental medicine, this is very similar to the notion of the Chiljeong Sang—the concept that excessive emotions accumulate and a lack of coping resources causes stress, which then affects the body and wounds the internal organs,^[3,4]^ as written in the Dongui Bogam.^[5,6]^ In addition, the ubiquity and stress of excessive emotions cause damage beyond the internal organs, because it has the greatest impact and influence on the “heart,” which is the center of the body.^[7,8]^ This is similar to the context of the Shim Ju Shin Ji (heart and brain) theory, in which the “heart” (meaning mind) controls the “brain” (meaning cognition) in Oriental medicine. Physiologically, the heart continuously transmits information to the cerebrum during each heartbeat, which affects information processing such as perception, cognition, and emotion, which are managed in the cerebrum.^[9,10]^ The vagus nerve in the heart controls the activation of the parasympathetic nervous system. The vagus nerve transmits information regarding the heart, such as heart rate, heart rhythm, and blood pressure,^[11]^ to the cerebrum through an afferent pathway.^[12]^ Thus, information regarding the heart, originating from the vagus nerve, is integrated in the nucleus solitarius of the brainstem through the afferent nerve pathway. In the nucleus solitarius, integrated afferent information is transmitted to the cerebral cortex via the limbic thalamus, hypothalamus, and amygdala. Information transmitted to the cerebral cortex is subjected to information processing in the brain, and then travels to the heart and major organs via the efferent pathway.^[13]^ Based on the information returning from the cerebrum, the heart regulates the sympathetic and parasympathetic nervous system of the autonomic nervous system to maintain the homeostasis of the human body, and is involved in human emotion, cognition, and behavioral responses.^[14,15]^ Therefore, rather than separate interpretations of the brain and heart, the heart should be considered as an organ that interacts with the brain. In Donguibogam, the concept of “heart” is recognized as 3 things: mind, heart, and center.^[16]^

This study is undertaken to scientifically clarify the theory of Shinju (mind and heart) (body's behavior), in which the “mind” (meaning heart) is involved in the “brain” (meaning cognition).^[17]^ To do this, we will analyze the correlation between heart–brain function by using 24-hour active electrocardiogram (ECG) and quantitative electroencephalogram (EEG) during meditation. Specifically, subjects will be randomly assigned to 1 of 2 meditations recalling resources or stress situations, to observe the relationship between changes in 24-hour active ECG and quantified EEG. We will monitor the connectivity of the heart and brain by measuring the ECG and quantitative EEG (qEEG) through this study for 24-hour Holter monitoring during meditation, and will thus obtain regarding heart rate variability (HRV) and biomarkers for blood pressure and qEEG. In addition, clinical outcomes through mediation interventions can be used in various areas, including stress treatment, pain reduction, and cognitive awareness therapy for emotional instability. Furthermore, based on the epistemology, it is expected that the clear scientific identification of psycho-brain correlations will be the basis of sustainable medical services in the field of psychiatric medicine in Korea.

## 2 Methods/design

This study protocol followed the Standard Protocol Items: Recommendations for Interventional Trials (SPIRIT) checklist, which enhanced the quality of the clinical trial.^[18]^

### 2.1 Objectives

To scientifically identify the theory that the heart controls the mentality related with the brain, it is going to study on the correlation between the heart (mind and heart) and the brain (
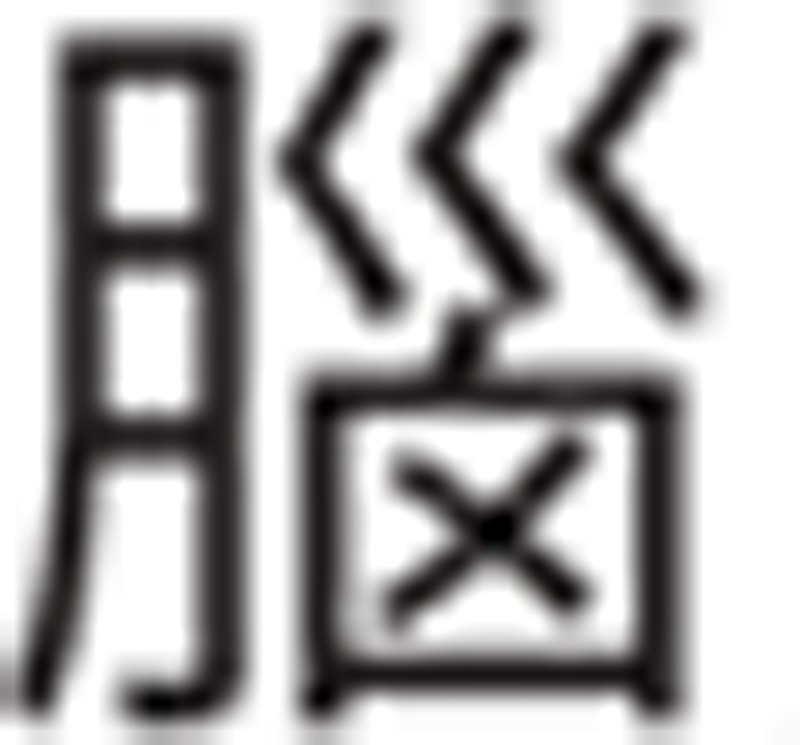
) function by measuring the qEEG^[19]^ and activity ECG for 24 hours under meditation.

### 2.2 Trial design and study setting

**Figure 1:**
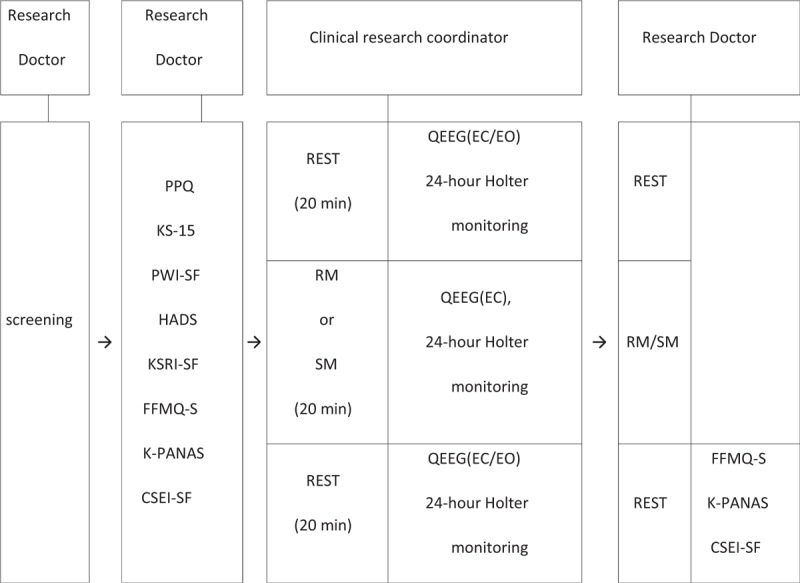
Summary of clinical trial design.

This clinical research will be conducted with subjects who provide voluntary consent and will be reviewed according to the inclusion or exclusion criteria of the clinical trial plan. Selected subjects will be assigned randomly into the test or active comparator group, according to precompleted random templates. The study design is as follows. This randomized, controlled, single-blinded, 2-armed, parallel, clinical trial will collect a total of 50 subjects, including 25 each for the test and active comparator groups. The subjects will be randomly allocated to a test group performing resource mindfulness (RM), and a control group performing stress mindfulness (SM), at a 1:1 ratio. The clinical trial consists of 3 stages, 20 minutes each (60 minutes total). The first and third stages are stable states. The second stage is divided into the test group and the active comparator group. The test group subjects will practice RM meditation and the active comparator group subjects will practice SM meditation. RM or SM meditation guide scripts will follow the M&L Psychotherapy Manual and the meditation guide will be directed by a professional practitioner trained by the M&L psychotherapist course. At stages 1 and 3, qEEG measurements will be taken for 10 minutes (5 minutes with eyes open and 5 minutes with eyes closed) from 5 minutes after the beginning of the stage. In the second stage, the qEEG measurements will be taken for 20 minutes, with eyes closed. The 24-hour Holter monitoring^[20]^ will be performed through stages 1, 2, and 3; HRV will be evaluated during each stage. The blood pressure and pulse will be measured before and after the second stage. All processes related with clinical trial including plans for collecting, assessing, reporting, and managing solicited and spontaneously reported adverse events and other unintended effects of trial interventions or trial conduct will be monitored and modified primarily by researcher director and peer researchers to ensure participants’ safety and research design's accuracy. A diagram and the time schedule of this study are shown in Table 1 and Fig. 1.

**Table 1:**
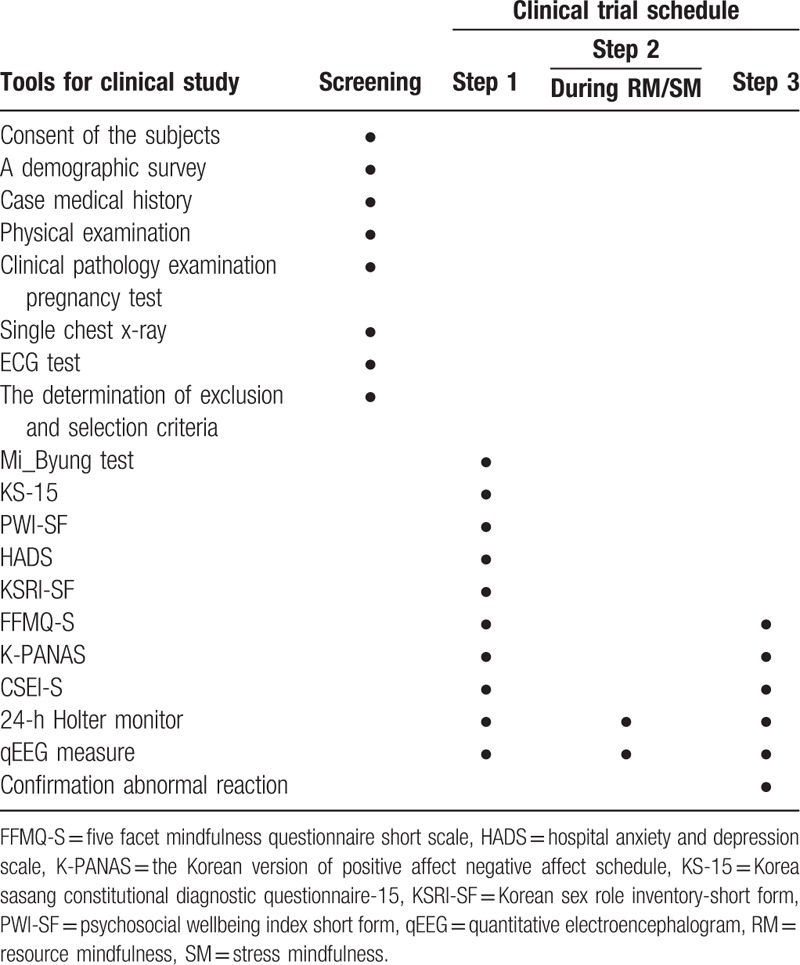
Time schedule of study.

### 2.3 Recruitment

Department of Neuropsychiatry of Korean Medicine & Inam Neuroscience Research Center, Wonkwang University Sanbon Hospital in Korea will recruit outpatients in the clinical practice setting. Clinical trial information will be posted on the bulletin board of each hospital and university, in online advertisements.

### 2.4 Eligibility criteria: inclusion criteria

1. Age: male and female individuals, age: 20 to 45 years
2. Individuals who do not consume excessive amounts of tobacco, tea, and coffee; who eat regularly; and who have a normal sleep pattern
3. Individuals who exhibit right-handedness
4. Individuals who voluntarily participate in the study, as indicated by their written consent (or that of a legal representative when the individual has limited ability to agree, but can still express willingness to participate)

### 2.5 Eligibility criteria: exclusion criteria

1. Individuals who demonstrate arrhythmia on EKG, or whose pulse rate per minute is outside of the range of 50 to 100
2. Individuals who have a severe physical illness or are medically unstable (as determined by the clinical trial conductor or another medical expert)
3. Individuals who have a congenital illness, psychological illness, central nervous problem, peripheral nervous problem, endocrinal illness, immunological disease, basal heart disease, liver disease, or renal disease
4. Individuals who have a neurological illness (eg, head trauma) or psychological history (eg, major depressive disorder, anxiety disorder, bipolar disorder, or schizophrenia)
5. Individuals with an intracardiac pacemaker inserted, or with a device that stimulates the brain, a ventricular peritoneal short circuit, or other artificial device inserted into the head
6. Individuals who take medicines that may affect the autonomic nervous system within 1 week before enrollment in the clinical trial (opiates, stimulants, tricyclic antidepressants, benzodiazepines, cardiac drugs such as beta-blockers, or renin-angiotensin-aldosterone system blockers such as angiotensin-converting enzyme inhibitors and angiotensin receptor blockers)
7. Individuals who smoke, drink alcohol, or ingest caffeine-containing products, within less than 2 hours before the clinical trial (eg, coffee, coffee milk, black tea, green tea, cocoa, colas/sodas, chocolate, caffeine-containing energy drinks)
8. Individuals who are pregnant or breast feeding
9. Individuals who participated in another clinical study within the prior 1 month or who are currently participating in other clinical studies
10. Individuals who exhibit difficultly participating in this study, as judged by the investigator

### 2.6 Subject withdrawal criteria

1. Violation of the inclusion criteria or fulfillment of the exclusion criteria
2. Serious adverse events that impede trial continuation
3. Withdrawal of consent by the subject or a legal representative
4. Do not receive 3 consecutive mindfulness meditation sessions
5. Violation of the clinical trial protocol by the investigator or the subject
6. Use of medications or treatments that can affect the results for clinical trial
7. Inappropriate progress as judged by the investigator

### 2.7 Randomization and allocation concealment

A randomization table will be created by an independent statistician using the balanced block.

#### 2.7.1 Randomization with Excel 2016

The randomization number allocated according to the assigned tables generated will be managed by the clinical trial manager and sent to each hospital. Consent will be obtained by a Korean Medicine doctor. If the participant voluntarily signs informed consent and meets the inclusion criteria, the clinical research coordinator (CRC) will sequentially allocate the participant to the treatment or control group according to the random number and record the subject identification code on the case recording sheet and the assignment table.

### 2.8 Blinding: single blind

Because of the study design, practitioners cannot be blinded. Only the participants will be blind. The assessor will be instructed to refrain from talking about the treatment to maintain blinding.

### 2.9 Intervention

#### 2.9.1 Study schedule

The study schedule is presented in Table 1. The study will include a screening phase, as a clinical step, the phase of before-meditation measurement, the phase of resource or stress mindfulness meditation, and the phase of postmeditation measurement. The participants will be recruited from the Wonkwang University Medical School Sanbon Hospital.

#### 2.9.2 Screening phase

At the screening phase, before conducting this clinical trial, the Korean oriental medical doctor (KMD) will explain to the subjects the contents of the “Instructions for obtaining subject consent” and confirm that the subject understands the contents, and that KMD must receive written permission that each subject is participating in the clinical trial of their own free will. And then, signing the informed consent form, the participants are selected as subjects by reviewing the selection criteria and exclusion criteria in accordance with the clinical trial plan. Selected subjects will fill in the demographic surveys, case medical history, physical examination, clinical pathology examination pregnancy test, single chest x-ray, ECG test, and will be assigned randomly into the test group or the active comparator group according to precompleted random templates, and after assigning a registration number, clinical trial will be conducted at the scheduled time. The subject identification codes will be recorded in the following manner:

- Provider Code: Wonkwang University Sanbon Hospital (WS)
- Agency Code – Test Year - Test Serial Number - Registered Order

(ex: WS-18-01-015: 15th registered patient in Wonkwang University Sanbon Hospital 1st clinical trial in 2018).

#### 2.9.3 Clinical phase

The clinical phases consist of 3 stages which prolong 20 minutes each (60 minutes total). The first and third stages are stable state. The second stage is to practice resource or stress mindfulness meditation. Before the clinical trial, at first stage, subjects will fill in the Pre clinic Phase (=Mibyung) Questionnaire, psychosocial wellbeing index short form,^[21]^ hospital anxiety and depression scale,^[22]^ Korean sex role inventory-short form,^[23]^ five facet mindfulness questionnaire short scale,^[24]^ the Korean version of positive affect negative affect schedule,^[25]^ the core seven emotions inventory-short form,^[26]^ and Korea Sasang Constitutional Diagnostic Questionnaire-15.^[27]^

At 1 and 3 stages, qEEG measurements were taken through 10 minutes (5 minutes with eyes open and 5 minutes with closed eyes) from 5 minutes after the beginning of the stage. In the second stage, the qEEG measurements were taken for 20 minutes with eyes closed. During 1, 2, and 3 stages, the 24-hour Holter monitoring was measured and HRV evaluated at each stage. The blood pressure and pulse were measured before and after the second stage.

After the clinical trial, at third stage, subjects filled the Five Facet Mindfulness Questionnaire Short Scale (FFMQ-S),^[24]^ the Korean version of positive affect negative affect schedule (K-PANAS),^[25]^ the core seven emotions inventory-short form (CSEI-SF),^[26]^ and confirmation abnormal reaction.

#### 2.9.4 Clinical trial phases: mindfulness meditation

Mindfulness is awareness training that observes what is happening in this moment as it is.^[28,29]^ It is a method to increase inner strength by examining the uncritical and nonjudging aspects of thoughts that arise from “deep inside,” while remaining at the bottom of consciousness. The resource or stress mindfulness guideline script will follow the M&L Psychotherapy Manual,^[30]^ and the Meditation Guide will be directed by trained mentors trained by the M&L Psychotherapist course. It should be noted that the drug injections or physician treatment that affect EEG and heart during clinical trials are prohibited.

#### 2.9.5 Test group intervention: resource mindfulness group

Resource mindfulness is to experience a safe and comfortable state by recalling the positive nourishment (resource) inside subject within the mindfulness state

#### 2.9.6 Active comparator group intervention: stress mindfulness group

Stress mindfulness is to experience emotions, feelings, memories, thoughts, and so on occurring within me when I recall a difficult situation inducing stress in the state of mindfulness.

### 2.10 Outcome measures: primary outcomes and measures

#### 2.10.1 qEEG

The EEG of the subjects will be measured in a comfortable, quiet, cool room, wearing an electro-cap (Electro-cap; Mitsar, Russia) with 21 surface electrodes placed in an international 10/20 system, for 5 minutes each in open-eye and closed-eye states. All electrode impedance will be less than 5 KΩ, based on the method of Ferree et al.^[31]^ The reference electrode will be located between Fz and Cz; the amplifier will be a Mitsar-EEG 201 machine (Mitsar, Russia). Artifacts of the recorded EEG, modified automatically, will be manually removed by the qEEG expert for a reliability measurement of 0.9 or better. Spectral analysis, coherence, and Low Resolution Brain Electromagnetic Tomography analysis will be performed with WinEEG for records with artifact-free EEG segments >2 minutes and split-half reliability >95%. Spectral analysis will use linked-ears reference montage and local average (Laplacian) montage.^[32,33]^ Linked-ears reference montage will be used as a general criterion; local average (Laplacian) montage can emphasize localized areas and minimize the overall effect of drugs on the brain.^[34]^ The absolute power and relative power will be calculated as delta (0.5–4 Hz), theta (4–8 Hz), alpha (8–12 Hz), and beta (12–20 Hz), respectively, using the absolute power and relative power. Neuro-guide will be used as the basis for all analyses; EEG analysis will be conducted by BCN, an expert KMD. In addition, the EEG results will be analyzed by KMD and CP after mentoring by a neuro feedback supervisor (BCN) and biofeedback specialist on Applied Psychophysiology in the United States.

#### 2.10.2 24-Hour Holter monitoring

**Figure 2:**
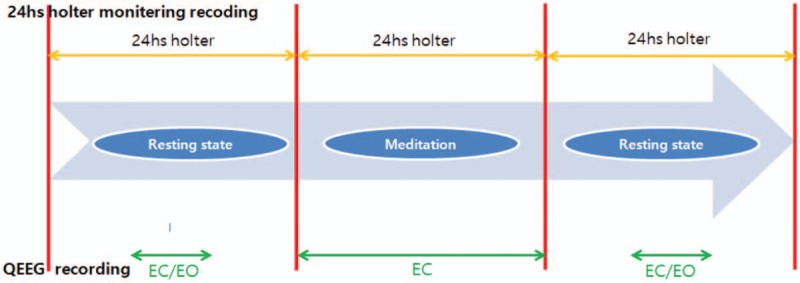
Flow chart of clinical trial design (qEEG and 24-hour Holter monitor). qEEG = quantitative electroencephalogram.

Heart rate variability is most commonly used to observe changes in the autonomic nervous system response.^[15]^ This allows quantitative measurement of the activity of the autonomic nervous system, is easily applicable, and is noninvasive to the subjects. In addition, it can continuously monitor the electrical activity of the heart to understand the inter-relationship of rhythm disorders, such as dizziness, chest pain, syncope, palpitations, and tachycardia, and to evaluate the mechanisms of arrhythmia, abnormal blood dynamics, and rhythm disorders. Therefore, in clinical practice, 24-hour Holter monitoring is a widely used method.^[35]^ The spectral density value (power spectral density, msec^2^) is calculated for the low-frequency component (0.04–0.15 Hz) and the high-frequency component (0.15–0.4 Hz) in frequency domain analysis (spectrum analysis). Then, the low-to-high frequency ratio (LF/HF) of the low-frequency spectrum is analyzed; it is compared with mean NN (mean of all coupling intervals between normal beats), SDNN (standard deviation of all normal RR intervals over 10 minutes), pNN50 (percent of differences between adjacent normal RR intervals of >50 ms during 10 minutes), and rMSSD (square root of the mean squared differences of successive NN intervals) in the time-domain analysis. Low-frequency domain values among indicators in the frequency domain represent the activity of the sympathetic nervous system. High-frequency domain values represent the activity of parasympathetic nerves. SDNN, rMSSD, and pNN50 are indicators of time domain that are used as indicators of the vagus nerve dominance pattern in the heart. In addition, active blood pressure monitoring can observe changes in blood pressure by simultaneously observing systolic and diastolic blood pressures before and after intervention, such as meditation. Biochemical markers such as insulin, fasting blood glucose and lipid levels, and changes in insulin resistance and blood lipid levels can be used to assess the associations of cardiovascular risk factors with biomarkers.^[36]^ The measurement and flow chart is Fig. 2.

#### 2.10.3 Others

Before the clinical trial, the subjects will complete questionnaires such as the constitutional diagnosis questionnaire, Korea Sasang Constitutional Diagnostic Questionnaire-15 (KS-15),^[27]^ hospital anxiety and depression scale (HADS),^[22]^ psychosocial wellbeing index short form (PWI-SF),^[21]^ KSRI-F,^[23]^ FFMQ-S,^[24]^ K-PANAS,^[25]^ and CSEI-SF.^[26]^ For the clinical trial, within 10 minutes before the start of phase 3, the subjects will complete the FFMQ-S,^[23]^ K-PANAS,^[24]^ and CSEI-SF^[25]^ questionnaires, and Confirmation abnormal reaction.

### 2.11 Safety assessment

Physical examinations will be performed, and vital signs and adverse events will be checked at every visit. To examine the safety of meditation, the KS-15,^[27]^ chest radiograph, electrocardiogram, clinical pathology ([hematology: hemoglobin, hematocrit, red blood cell count, white blood cell count, platelet count, white blood cell percentage], blood chemistry, gamma-glutamyl transpeptidase, alkaline phosphatase, blood urea nitrogen, creatinine, T-bilirubin, and glucose], pregnancy test (urine HCG test), and infectious disease test by KMD and CP at the screening visit will be conducted by KMD or CP. physicians with ≥5 years of clinical experience will oversee the clinical evaluations.

During the clinical trial, any adverse event will be recorded and assessed in case report form (CRF). It will be calculated to the adverse event occurrence ratio. Adverse events associated with the meditation intervention will be monitored and appropriately handled by KMD and CP.

### 2.12 Data management and quality control

The CRC will collect data on a paper CRF. Records including medical records, questionnaires, consent forms, and other clinical trial relevant papers will be stored at each site in a locked cabinet or a password-protected computer that can be accessed only by an authorized individual and kept confidential, even when the results of the clinical trial are published. However, quester, monitor, and inspectors related to this clinical trial can view each subject's records for the purpose of monitoring and checking this clinical trial and management of the subject's progress. Additionally, all documents related to this clinical trial, such as case histories, will be recorded and distinguished by using each subject's identification code (typically the subject's initials), but not by using any subject's name. After completion of the outcome report, the research will be kept for 5 years from the end of the clinical trial.

### 2.13 Sample size calculation

Based on the clinical trial study, the number of subjects was calculated as a minimum of 25 subjects each in the test and control groups, considering a 10% dropout rate. Based on previous studies,^[37,38]^ using the G∗power program, the total effect size was set at 1.0, and the total number of samples in the 90% confidence interval (CI) was set at 46 subjects. Considering a dropout rate of 10%, 50 participants must be recruited to collect sufficient data for statistical analysis and interpretation.

### 2.14 Statistical and analytical plans

To analyze the results of this clinical trial, the statistical software program SPSS 23.0 will be used to verify the homogeneity of each subject. The specific statistical analysis procedure is as follows. First, descriptive analysis (mean, frequency, SD) will be performed on the measured variables. Second, compared with baseline, nonparametric Wilcoxon sign^[39]^ and Wilcoxon matched-pair signed-ranks tests^[40]^ will be performed, according to the test group (RM) and the control group (SM). Third, a verification of differences will be performed between qEEG,^[19]^ 24-hour Holter monitoring,^[20]^ FFMQ-S,^[24]^ CSEI-SF,^[26]^ and K-PANAS,^[25]^ according to the baseline test and control groups. Finally, a nonparametric Spearman correlation analysis will be conducted on the basis of the quantitative brain wave (qEEG^[19]^), 24-hour Holter monitoring,^[20]^ FFMQ-S,^[24]^ CSEI-SF,^[26]^ and K-PANAS^[25]^ scales.

### 2.15 Ethics approval and consent to participate

This study was approved by Wonkwang University Sanbon Hospital (WMCSB 201802-09). The protocol for this study has been registered at the Clinical Research Information Service (CRIS; https://cris.nih.go.kr) The clinical trial registration number is KCT0003012. The final protocol version is 2.3 and dated July 21, 2018. A KMD in Wonkwang University Sanbon Hospital will ask participants who have decided to voluntarily enroll in the study to submit written consent. All data obtained from the subjects will be secured. Hard copies will be stored in double-locked locations in the laboratory of the hospital, and soft copies will be saved in memory devices not connected to the internet, also stored in double-locked locations. The corresponding author will have access to the final dataset. All subjects will be respected and protected, in accordance with the Declaration of Helsinki, and related laws and regulations. The results of this study will be published in a peer-reviewed journal.

## 3 Discussion

This protocol was developed to analyze the relationship between brain and heart through RM and SM. There are some limitations to this present study. First, the participating institutions do not represent all clinical institutions in Korea, because this is limited to single institution. Second, because the participant age in this study is limited to the younger generation, it is not possible to generalize, with respect to age-related outcomes. Third, this study may exhibit difficulty in acquiring all of each subject's personal information because the measurement and intervention of the project is performed in 1 day.

However, the data obtained in this study relating to the brain and heart can be used as basic data and reference data for future brain–heart research. In addition, we will expand the potential impact of oriental medical technology evaluation as evidence-based medicine, establish a scientific basis for clinical trials theory based on clinical data, generate a clinical model and quantitative index for the evaluation of meditation therapy, and identify effects of meditation therapy. Finally, this study will be the first to scientifically measure and analyze the brain–heart relationship in Korea. Therefore, the results of this study will help to gain information and build medical systems for enhanced medical intervention in all diseases that are related to brain and heart.

### 3.1 Trial status

This trial is currently recruiting participants. Recruitment began on July 23, 2018. We expect the recruitment phase to be complete by October 31, 2018.

## Author contributions

**Conceptualization:** Moon Joo Cheong, Sung Eun Son, Hyung Won Kang, Kwang-Ho Bae, Yeonseok Kang.

**Data curation:** Moon Joo Cheong, Hyung Won Kang.

**Formal analysis:** Moon Joo Cheong, Hyung Won Kang.

**Funding acquisition:** Hyung Won Kang.

**Investigation:** Sung Eun Son, Youngseop Lee, Eun Mi Lee, Go-Eun Lee, Joo-Hee Seo, Hee-Wook Weon, Ji-Yoon Lee, Yeoung-Su Lyu.

**Methodology:** Moon Joo Cheong, Sung Eun Son, Hyung Won Kang.

**Project administration:** Hyung Won Kang.

**Resources:** Youngseop Lee, Kwang-Ho Bae, Yeonseok Kang.

**Supervision:** Hyung Won Kang.

**Writing – original draft:** Moon Joo Cheong, Sung Eun Son, Hyung Won Kang.

**Writing – review & editing:** Moon Joo Cheong, Hyung Won Kang.

Hyung Won Kang orcid: 0000-0001-6497-0100.
